# Unique PFK regulatory property from some mosquito vectors of disease, and from *Drosophila melanogaster*

**DOI:** 10.1186/s13071-016-1391-y

**Published:** 2016-02-25

**Authors:** Rodrigo Dutra Nunes, Nelilma Correia Romeiro, Hugo Tremonte De Carvalho, Jean Ribeiro Moreira, Mauro Sola-Penna, Mário Alberto C. Silva-Neto, Glória Regina Cardoso Braz

**Affiliations:** Instituto de Bioquímica Médica Leopoldo de Meis, Universidade Federal do Rio de Janeiro, Rio de Janeiro, RJ Brazil; Departamento de Química Orgânica, Instituto de Química, Universidade Federal do Rio de Janeiro, Rio de Janeiro, RJ Brazil; NUPEM-Macaé, Universidade Federal do Rio de Janeiro, Rio de Janeiro, RJ Brazil; Departamento de Bioquímica, Instituto de Química, Universidade Federal do Rio de Janeiro, Riode Janeiro, RJ Brazil; Faculdade de Farmácia, Universidade Federal do Rio de Janeiro, Rio de Janeiro, RJ Brazil; Instituto Nacional de Ciência e Tecnologia em Entomologia Molecular, Rio de Janeiro, Brazil

## Abstract

**Background:**

Arthropod-borne diseases are some of the most rapidly spreading diseases. Reducing the vector population is currently the only effective way to reduce case numbers. Central metabolic pathways are potential targets to control vector populations, but have not been well explored to this aim. The information available on energy metabolism, as a way to control lifespan and dispersion through flight of dipteran vectors, is inadequate.

**Methods:**

Phosphofructokinase (PFK) activity was measured in the presence of both of its substrates, fructose-6-phosphate (F6P) and ATP, as well as some allosteric effectors: Fructose- 2,6 – bisphosphate (F2, 6BP), citrate and AMP. *Aedes aegypti* phosphofructokinase sequence (AaPFK) was aligned with many other insects and also vertebrate sequences. A 3D AaPFK model was produced and docking experiments were performed with AMP and citrate.

**Results:**

The kinetic parameters of AaPFK were determined for both substrates: F6P (V = 4.47 ± 0.15 μmol of F1, 6BP/min, K_0.5_ = 1.48 ± 0.22 mM) and ATP (V = 4.73 ± 0.57 μmol of F1, 6BP/min, K_0.5_ = 0.43 ± 0.10 mM). F2,6P was a powerful activator of AaPFK, even at low ATP concentrations. AaPFK inhibition by ATP was not enhanced by citrate, consistent with observations in other insects. After examining the sequence alignment of insect and non-insect PFKs, the hypothesis is that a modification of the citrate binding site is responsible for this unique behavior. AMP, a well-known positive effector of PFK, was not capable of reverting ATP inhibition. *Aedes, Anopheles and Culex* are dengue, malaria and filariasis vectors, respectively, and are shown to have this distinct characteristic in phosphofructokinase control. The alignment of several insect PFKs suggested a difference in the AMP binding site and a significant change in local charges, which introduces a highly negative charge in this part of the protein, making the binding of AMP unlikely. This hypothesis was supported by 3D modeling of PFK with AMP docking, which suggested that the AMP molecule binds in a reverse orientation due to the electrostatic environment. The present findings imply a potential new way to control PFK activity and are a unique feature of these Diptera*.*

**Conclusions:**

The present findings provide the first molecular explanation for citrate insensitivity in insect PFKs, as well as demonstrating for the first time AMP insensitivity in dipterans. It also identified a potential target for novel insecticides for the control of arthropod-borne diseases.

**Electronic supplementary material:**

The online version of this article (doi:10.1186/s13071-016-1391-y) contains supplementary material, which is available to authorized users.

## Background

Glycolysis is the most conserved metabolic pathway and it is found in almost all forms of life. It has ten catalysed reactions to the conversion of glucose to pyruvate. The flow rate of this pathway is regulated by the substrates availability and the allosteric control of the enzymes that catalyse reactions irreversible *in vivo*, which are hexokinase (EC: 2.7.1.1), phosphofructokinase (EC: 2.7.1.11) and pyruvate kinase (EC: 2.7.1.40). Because of PFK’s important role as a regulatory enzyme of this pathway its properties have been the target of study in several organisms.

Since 1984, it has been known that eukaryotic phosphofructokinase (PFK, EC 12.7.1.11) is more than twice the size of bacterial PFK [1]. It was previously proposed that this is a result of a tandem gene duplication followed by fusion. It was also suggested that the catalytic site of the new enzyme remained in the amino terminal segment. However, the corresponding site in the carboxylic segment of the new protein evolved to become the effector-binding site. Site directed mutagenesis studies, and other techniques seeking to confirm this hypothesis, have been published and the evidence supporting it is readily available [2–5].

In most organisms, PFK is inhibited by physiological concentrations of one of its substrates, ATP. Thus, PFK activity achieves a significant rate only when the inhibitory effect of ATP is counteracted by at least one of PFK’s several activators. In vertebrates ADP, AMP and F2, 6P are the most common PFK activators [3]. In addition to ATP, citrate also inhibits PFK. Newsholme and co-workers [6] investigated the effect of citrate on PFK activity in muscle homogenates from nine insects and in the cerebral ganglion of the locust. Citrate does not inhibit PFK in any of these organisms. Other investigations have confirmed the absence of citrate inhibition in insects [7, 8], but no other distinguishing regulatory features of insect PFK has been demonstrated. Although dipterans are of major importance in epidemiology, no research on the regulatory properties of PFK from any flight vector has been published to date.

*Aedes aegypti* is the vector of dengue, the most rapidly spreading vector borne disease, as well of many other arboviruses like Chikungunya, Yellow fever virus and Zika virus. There are an estimated 50 million dengue infections annually and approximately 2.5 billion people live in dengue endemic countries. There are currently no dengue-specific drugs nor well tested vaccines against this disease [9]. Reducing the vector population is currently the only effective way to reduce case numbers and consequent fatality rates. However, insect resistance to the insecticides that are currently available is hampering this effort. Thus, it would be of great epidemiological significance to find ways of targeting diverse aspects of vector biology in order to contain the spread of Dengue. Dengue virus takes 8 - 12 days to propagate inside the mosquito and to reach the salivary glands [10]. Feeding on carbohydrates after a blood meal significantly increases the life span and dispersion of female mosquitoes [11, 12]. Therefore, the study of sugar utilization by mosquitoes should no longer be neglected. Only the female is capable of feeding on blood and is therefore a disease vector. This, together with the once accepted hypothesis that, in nature, female mosquitoes rarely feed on sugar [13], may explain the lack of available studies about the glycolytic pathway in this insect. Nevertheless it was shown [14] that this hypothesis was based on an inaccurate method used to quantify carbohydrates in field-captured mosquitoes. Measurable amounts of fructose, an indication of the ingestion of plant sugars by the mosquito, in Brazilian field-captured mosquitoes were found [14]. To gain information on the control of the glycolytic pathway in *A. aegypti* and, perhaps, to find a new target for controlling mosquito populations or longevity, this study investigated the kinetic properties of PFK, which is one of the enzymes that physiologically regulates glycolytic flux in all of the organisms studied to date. Unique patterns of citrate and AMP insensitivity in this insect group were found. Herein, molecular explanations are proposed, based on molecular 3D modeling and docking experiments, for the relevance of such findings in Diptera and their potential use as novel targets to block disease transmission.

## Methods

### Ethics Statement

All animal care and experimental protocols were conducted in accordance with the guidelines of the institutional care and use committee (Comissão de Avaliação do Uso de Animais em Pesquisa da Universidade Federal do Rio de Janeiro, CAUAP-UFRJ) and the NIH Guide for the Care and Use of Laboratory Animals (ISBN 0-309-05377-3). The protocols received registry number 115/13 from the Animal Ethics Committee (Comissão de Ética no Uso de Animais, CEUA).

### Mosquito Rearing and Sample Preparation

*A. aegypti* (Red Eye strain) were reared at 28 ± 2 °C and 80 ± 5 % humidity on a 12 h light-dark cycle under standard laboratory conditions. *Culex quinquefasciatus* and *Anopheles aquasalis* mosquitoes were kindly provided by Dr. Denise Valle from the Laboratory of Physiology and Control of Arthropod Vectors/Oswaldo Cruz Foundation. *Drosophila melanogaster* flies were provided by Dr. Blanche Christine Pires de Bitner-Mathé Leal from the Laboratory of Population Genetics of *Drosophila*, Federal University of Rio de Janeiro. Groups of 10 insects were homogenized in 150 μL of 50 mM Tris-HCl pH 7.4, 0.15 M NaCl. The extract was centrifuged at 130,000 x g, at 30 psi, for 60 min (Beckman Airfuge, Beckman Instruments Inc., CA, USA). An aliquot of the supernatant (100 μL) was collected and used for enzyme activity measurements.

### Phosphofructokinase activity

PFK activity was measured as described previously [15, 16]. The reaction media contained 50 mM Tris-HCl pH 7.4, 1.8 mM (NH_4_)_2_SO_4_, 2 mM NADH, 5 mM MgCl_2_ and, unless otherwise stated, 1 mM ATP and F6P, as well as the coupled enzymes aldolase (4 U/mL), triose phosphate isomerase (4 U/mL) and α-glycerophosphate dehydrogenase (4 U/mL). The reaction was started by the addition of 10 μL of homogenate, which contained approximately 10 μg of protein, to the reaction mixture. The oxidation of NADH was monitored spectrophotometrically for 60 min at 340 nm, and the linear phase was used to calculate the rate of the enzyme-catalyzed reaction. An extinction coefficient of 6.220 × 10^6^ M^-1^ cm^-1^ for NADH was used for the indirect calculation of the F1, 6BP production. The initial velocity (V_0_) of NADH oxidation, without added substrate, was subtracted from all activities measured. The initial reaction rate is expressed as U/mg protein, with one U being considered as the formation of 1 μmol F1, 6BP per minute. Protein concentration was measured by the Lowry method [17], using BSA as a standard. A scan of the activity was performed in the pH range of 6.0 to 9.0 in order to determine the optimum pH, which was in the range 8.0-8.5. Nevertheless pH 7.4 was used as this was the pH that was previously described to be the one where the enzyme is most sensitive to allosteric regulation [8].

### Kinetic and statistical analysis

The Kinetic parameters of maximal velocity (Vmax), Hill’s coefficient (h), Kprime (K_0.5_^h) and the constant of inhibition (Ki) for the substrate curves were calculated by non-linear regression using the software Sigma-Plot 10 (Systat, CA, USA). Values are the mean ± standard errors of the parameters, calculated by fitting the curves to the previously described equations [8].

### Sequence alignment

PFK sequences from several organisms were obtained from NCBI (http://www.ncbi.nlm.nih.gov/protein/) [18]. Acession numbers: ORYCU-M XP_002723486; HOMSA-M NP_001160159; ORYCU-C NP_001076217; MUSMU-C NP_062677; AEDAE XP_001652300; CULPI XP_001850333; ANOGA XP_564589; DROME NP_724891; DROVI XP_002050868; DROPE XP_001361103; ACYPI XP_001950251; TRICA XP_966779; PEDHU XP_002433151; BOMTE XP_012168289; 3o8L:A (PDB) and RPRC RPRC011744 (Vector Base). Multi-sequence alignment analysis was performed using Clustal W2 [19].

### Molecular modeling of PFK

The 3D comparative models of *A. aegypti* and human PFKs were constructed using the NCBI amino acid sequences with code numbers: NP_000280.1 (*Homo sapiens*) and EAT41468.1 (*A. aegypti*). In the course of this work, crystal structures of PFK from *Pichia pastoris* (PDB ID 3OPY) and rabbit skeletal muscle (PDB ID 3O8L) were deposited in the Protein Data Bank [20–22]. Because of its high sequence similarity, the structure of PFK from rabbit skeletal muscle seemed most suitable for our structural analysis. PDB entry 3O8L was chosen as the template for the construction of the 3D comparative models of PFK, using the CPH models 3.2 server (http://www.cbs.dtu.dk/services/CPHmodels/) [23]. In this work, structural verification of the PFK models was performed using the protein analysis tools in the Structural Analysis and Verification Server (http://services.mbi.ucla.edu/SAVES/), which provides several tools for this purpose, such as the Ramachandran plot and Verify 3D tools. Visual inspections of the three-dimensional models were made using PyMOL version 0.99 (http://www.pymol.org/). Electrostatic potential calculations were performed with PDB2QPR and APBS [24, 25] and volume calculations were performed with Surfnet Tools available in the UCSF Chimera software [26].

### Docking Studies

The structure of AMP was extracted from the X-ray structure of PFK from *Pyrococcus horikoshii* (PDB ID 3DRW) [27] and hydrogen atoms were added with Spartan for Windows v.8 (Wavefunction Inc.). AMP geometry optimization was done using the semi-empirical method AM1 [28]. Chemscore_Kinase fitness function available in GOLD docking software version 4.1.2 [29] was used to score the docked AMP structures. The binding site was defined in the GOLD software as being all atoms within a 10 Å radius from Lys475 or Lys487, in the human or *A. aegypti* forms of PFK, respectively. The protein-ligand complexes with the most favourable fitness score values among the top scored complexes were used for further visual inspection. During the automated docking process, GOLD applies a flexible ligand docking methodology in which stored conformations are posed in the protein binding site, taking into account the full acyclic flexibility and partial cycle flexibility.

## Results and Discussion

### Determination of optimum pH and Kinetic Parameters of AaPFK

PFK is a well-studied enzyme, but its regulatory properties are not fully understood. Most of the research about its regulatory properties has been carried out in mammals or in microorganisms, because of the ease of its purification directly from these sources. Attempts to purify active recombinant enzymes have often failed due to the multimeric structure of the enzyme that usually promotes its aggregation in an inactive form. Also, this enzyme has catalytic activity only in its tetrameric form, having almost no activity as a dimer and no detectable activity as a monomer. This aggregation also hampers attempts to crystallize PFK and resolve its crystal structure [21]. However, according to the author, a useful recombinant protein was only obtained when the last 18 amino acids were removed. Nevertheless, recombinant mammalian PFK has recently been crystallized [30].

Several studies with PFK in insect models have been conducted [8, 31, 32]. In those studies, PFK activity was measured under non-standardized conditions, making it difficult to compare the results obtained by these authors. Herein presented is the characterization of AaPFK activity, measured using insect extracts.

**Fig. 1:**
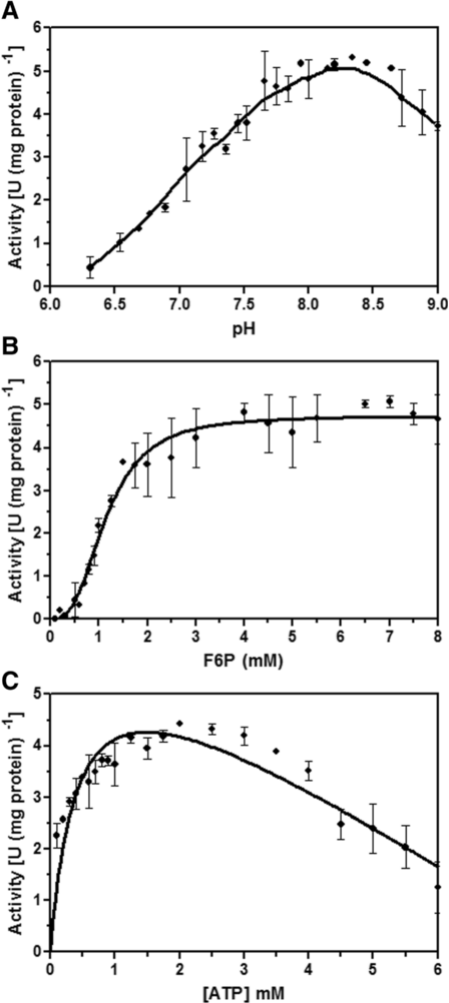
AaPFK activity as a function of pH (**a**), F6P (**b**) and ATP (**c**) concentrations. The PFK activity of *Aedes aegypti* was measured in the presence of 1 mM ATP and 1 mM F6P or at the concentrations indicated, at pH 8.0 unless otherwise specified. Values are means ± SEM of three independent experiments

First, the activity was measured at several pH values in the range of 6.2 to 9.0. A bell-shape curve was obtained (Fig. 1A) and AaPFK maximal activity was observed in the pH 8-8.5 range. All other assays presented here were performed at pH 7.4, as the objective was to measure physiological PFK activity and this is the pH where allosteric regulation AaPFK is better evidenced (data not shown).

The kinetic parameters of AaPFK were determined by measuring the initial reaction rate as a function of substrate concentration varying the concentrations of each of the enzyme substrates, F6P and ATP, one at a time. The initial reaction rate is expressed as U/mg protein, with 1 U being the formation of one μmol F1, 6BP per minute. Figures 1B and C show the saturation curves of AaPFK by its substrates F6P and ATP, respectively. The reaction rate shows cooperativity in F6P binding. The experimental data were best fitted to the parameters of the equation previously described [8] and were used to assemble Table 1. The kinetic parameters obtained confirm that at concentrations above 2 mM, ATP becomes a potent inhibitor of PFK activity. Table 1 indicates that AaPFK has cooperativity towards F6P and ATP, as expected [8]. Assays at very low non-physiological ATP concentrations would be necessary to corroborate the cooperativity of this enzyme in this part of the saturation curve. Because the aim of this work was to gain knowledge on the *in vivo* control of AaPFK, the enzyme was not assayed at such low, non physiological ATP concentrations. Additionally, the assays were conducted using crude enzyme preparations, which does not favour precise kinetic parameter determination, but allows better *in vivo* conclusions.

**Table 1:** Kinetic parameters of *Aedes aegypti* PFK upon addition of its substrates F6P and ATP

	F6P	ATP
Parameter	Activity [U (mg protein) ^-1^]	Activity [U (mg protein) ^-1^]
Vmax	4.741 ± 0.1462	4.727 ± 0.568
h	2.798 ± 0.3939	0.741 ± 0.203
Kprime	1.485 ± 0.2192	0.429 ± 0.100
K_i_	-----	4.536 ± 0.178

PFK activity was measured at pH = 7.4, F6P and ATP range 0.1–8.0 mM. Kinetic parameters for the substrate curves were calculated by non-linear regression using the software Sigma-Plot 10 (Systat, CA, USA). Values are the mean ± SEM. Data from three independent experiments were used to fit through the equations described in the Methods. Vmax - maximal velocity; h – Hill’s coeficient; Kprime – K_0.5_^h, Ki – constant of inhibition

### Regulation of AaPFK by negative effectors

PFK is tightly regulated in all organisms. The rate of glycolysis is determined by a balance between the rate-limiting reactions of the pathway towards sugar degradation and the rate of the reverse reactions. This balance is the result of interactions of these enzymes with substrates and several allosteric effectors.

As shown in Fig. 1C, physiological concentrations of ATP strongly inhibit AaPFK activity. Among the known PFK inhibitors, citrate is known to enhance the inhibitory effect of ATP on mammalian PFKs [3]. It has been reported that citrate does not exhibit inhibitory effects on insect PFKs. However, this lack of citrate inhibition was never properly explained [6]. Even 30 years after this original finding, which has since been extensively reported, no biochemical explanation has been provided for the insensitivity of insect PFK to citrate [3, 6, 7].

Here we show that AaPFK activity, at several sodium citrate concentrations, exhibits no change in activity, either in the presence of inhibitory ATP concentrations or at non-inhibitory ATP concentrations (Table 2). Because this extensively reported phenomenon remains unexplained, we investigated whether the lack of citrate effect on insect PFK could be due to modifications in the citrate binding site or by other structural changes in the protein.

**Table 2:** Effect of citrate on *Aedes aegypti* PFK activity

Citrate (mM)	Activity [U (mg protein) ^-1^]	
	ATP (1 mM)	ATP (5 mM)
0	4.02 ± 0.26	2.50 ± 0.13
0.25-8.0	4.01 ± 0.40	2.44 ± 0.19

PFK activity was measured at pH = 7.4, 1 mM F6P at low and high ATP concentrations, without (first row) and with added citrate. The assay was conducted at eight different citrate concentrations in the range of 0.25 – 8 mM. Values are means ± SEM of four independent experiments

**Fig. 2:**
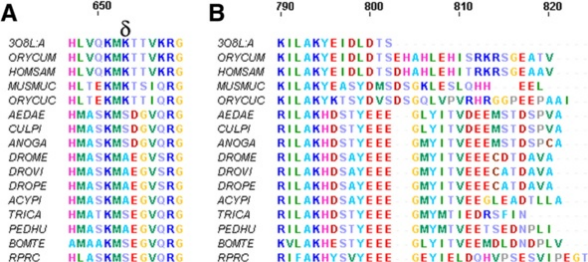
Alignment of amino acid sequences for the citrate binding site of several PFKs. The putative residues assigned to the binding of citrate and in the vicinity of this site where the greatest differences are observed are indicated by **δ**. The sequences shown are from: *Oryctolagus cuniculus* (ORYCU), *Mus musculus* (MUSMU), *Homo sapiens* (HOMSA), *Aedes aegypti*, (AEDAE), *Culex quinquefasciatus* (CULPI), *Anopheles gambiae*, ANOGA), *Drosophila melanogaser* (DROME), *Drosophila virilis* (DROVI), *Drosophila pseudoobscura* (DROPE), *Acyrthosiphon pisum,* (ACYPI), *Tribolium castaneum*, (TRICA), *Pediculus humanus corporis* (PEDHU), *Bombus terretris* (BOMTE), *Rhodnius prolixus* (RPRC)

To study this hypothesis the AaPFK amino acid sequence was aligned to the PFK sequences from other insects and to some mammalian sequences (Additional file 1: Figure S1). As expected for insects, only one isoform of PFK was found in the *A. aegypti* proteome. The amino acids that are essential for the interaction of PFK with several ligands according to Kemp and Gunasekera are marked [2]. The conservation of the amino acids required for the citrate inhibitory effect was examined (Additional file 1: Figure S1, symbol **δ**), and only two changes were observed. First, Lys557 (human PFK numbering) is replaced by Arg in all of the insect sequences examined, which is a conservative substitution. Second, Lys617 (human PFK numbering), an amino acid bearing a positively charged side chain, is replaced by either Ser or Ala, neutral amino acids, in all insect sequences examined (Fig. 2A, symbol **δ)**. Near this substitution, a neutral Threonine (Thr618 - human PFK numbering) in mammals is substituted by a negatively charged residue in all insects (Glu or Asp) (Fig. 2A). Taken together, these substitutions could be partially responsible for the lower affinity of the negatively charged citrate molecule for the protein binding site, rendering the enzyme insensitive to citrate inhibition.

It is also known that citrate does not bind to mammalian PFK if the last 30 amino acids of its sequence are removed by enzymatic proteolysis [3, 33]. Fig. 2 shows the alignment of the carboxyl terminal region of several PFKs. A high conservation of amino acid sequence is found in animal PFKs. Nevertheless, the high conservation of the insect carboxyl-terminal region amongst insects is different from that of vertebrates. In insects, this region is characterized by a high number of negatively charged amino acids (Fig. 2B). Martínez-Costa *et al*. have also shown [3] that replacing Leu767 and Glu768 of the M-type isoform of human PFK (PFK-M) with glycine prevents citrate from binding to the enzyme. Although these residues have never been implicated in citrate binding to the enzyme, their relevance to citrate inhibitory effect was demonstrated. It is possible that these residues are important to guide the binding of citrate into PFK. These residues are not conserved in insect PFKs (Fig. 2B), which could be an additional reason for its insensitivity to citrate regulation.

**Fig. 3:**
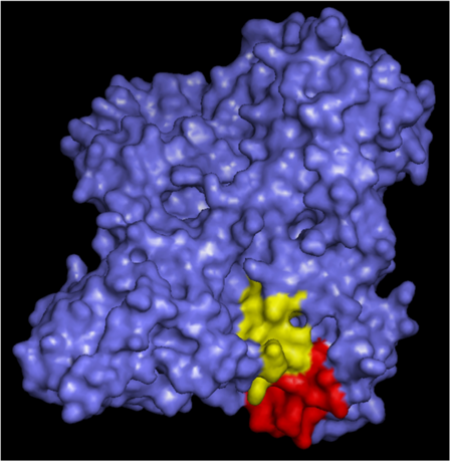
3D representation of rabbit PFK-M. Molecular surface representations of rabbit skeletal muscle PFK (PDB ID 3O8L) emphasizing the citrate binding site (*red*) and the last 12 amino acids in the C-terminal tail (*yellow*)

The highly negatively charged C-terminal terminus found in insects may explain why correct guidance of citrate by this region of the insect protein is unlikely. This hypothesis would be supported if the 3D structure of the protein indicates that these residues are involved in correct binding. Unfortunately, 3D comparative modeling of the C-terminal tail is not possible at this time because of a lack of template. Until recently, there was no 3D structure of a eukaryotic PFK available as a template for citrate docking analysis, except for *Trypanosoma brucei*, which has only 487 amino acid residues and lacks the residues in question. In 2011, a crystal structure of PFK from rabbit skeletal muscle (PDB code *3O8L*) [21] was deposited into the PDB. Unfortunately, the recombinant form of that enzyme is missing the final 18 amino acids. Nevertheless, the C-terminal residues in the crystallized protein show that the C-terminal tail is very close to the pocket where citrate binds (Fig. 3). Very recently, other mammalian crystal structure of PFK were published, including the one from human platelets [30], which has some differences from the muscle, so it was not used in this work. Altogether these observations reinforce our proposition that the negative nature of the carboxy terminus of insect PFKs is one of, if not the major, cause for its insensitivity to citrate.

This C-terminal extension of the mammalian enzyme, when compared to the bacterial enzyme, [33] seems to have evolved in order to provide mammals with this control, which is essential for coordinating carbohydrate and fatty acid metabolism. In mammals, the presence of citrate in the cytoplasm, where glycolysis occurs, indicates that fatty acid synthesis is underway. To make this possible, the cell must have fulfilled its ATP requirements, whilst still having excess carbon building blocks available that cannot be incorporated into glycogen. When this excess ATP is observed, respiration slows down and NADH concentrations inside the mitochondria rise, which inhibits isocitrate dehydrogenase. This slows the citric acid cycle and allows citrate concentrations to build up inside the mitochondria, causing citrate to be pumped out to the cytoplasm. Citrate does not accumulate in the cytoplasm, but is readily converted to acetyl-CoA and oxaloacetate by citrate lyase and supplies the carbon source for fatty acid biosynthesis. The cytoplasmic levels of citrate build up in the cytoplasm and inhibit PFK only if the products of cytoplasmic citrate lyase are not rapidly consumed [34, 35]. It seems that this control is not as essential in insects because sugar is their major food source, including females from anautogenous species. Thus, these animals need to perform glycolysis and fatty acid synthesis at the same time. In the case of female mosquitoes, part of the sugar ingested before a blood meal is converted to lipid that accumulates in oocytes. This lipid accumulation minimizes follicular reabsorption, thus increasing the fertility rate [36].

### Regulation of AaPFK by positive effectors

F2, 6BP is one of the most potent activators of PFK. As expected, F2, 6BP activated AaPFK even at a concentration of ATP (1 mM) that is not inhibitory (Additional file 2: Figure S2). The activation factor shown for F2, 6BP is lower than the one described for other organisms [5]. This is probably due to the fact that the enzyme was not purified prior to the assay, so the presence of some endogenous F2, 6BP could mask its effect, as F2, 6BP activates PFK at concentrations below 1 μM. Furthermore, some 6-phosphofructo-2-kinase/fructose-2,6-bisphosphatase-like activity must be present in the homogenate, and the conversion of added F6P to F2, 6BP is likely to occur because both enzymes share the same substrates.

**Fig. 4:**
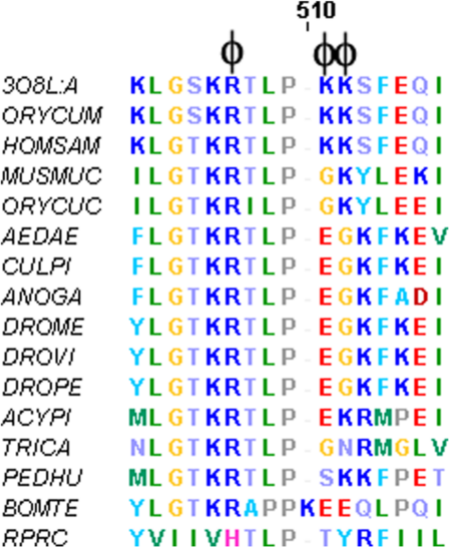
Alignment of amino acid sequences of the AMP binding site of several PFKs. The putative residue assigned to the binding of AMP and its vicinity is indicated by **ϕ**. The sequences shown are from: *Oryctolagus cuniculus* (ORYCU), *Mus musculus* (MUSMU), *Homo sapiens* (HOMSA), *Aedes aegypti*, (AEDAE), *Culex quinquefasciatus* (CULPI), *Anopheles gambiae*, ANOGA), *Drosophila melanogaser* (DROME), *Drosophila virilis* (DROVI), *Drosophila pseudoobscura* (DROPE), *Acyrthosiphon pisum,* (ACYPI), *Tribolium castaneum*, (TRICA), *Pediculus humanus corporis* (PEDHU), *Bombus terretris* (BOMTE), *Rhodnius prolixus* (RPRC)

Another well-known effector of PFK is AMP. The effect of AMP as an allosteric activator of vertebrate PFK is undisputable [34]. In insects, this effect has only been reported in bees and kissing bugs [8, 37]. Besides these reports, no other references regarding AMP affecting PFK kinetic properties in insects were found. A specific region of PFKs close to residues Glu485, Gly486 and Lys487 (AaPFK numbering) have been reported as being important for AMP binding and activation of PFK in mammals [2]. In order to see if there is an alteration in the corresponding residues in *A. aegypti* and other insects, the sequences from several organisms were aligned to the one described by Kemp and Gunasekera [2] (Additional file 1: Figure S1). There is at least one spot assigned to AMP binding in mammals that is modified in AaPFK and also in all other known dipteran PFK sequences examined. This substitution is not present in the sequences of PFKs from other orders of insects but Hymenoptera. Figure 4 shows in more detail the substitutions in the AMP binding site of the human 475 and 476 positively charged lysine residues by glutamic acid and glycine in AaPFK. These replacements cause a profound change in the electrostatic properties of the AMP binding site. The resulting negative local charge could repel AMP, a negatively charged molecule, preventing AMP from docking at this site.

The comparative analysis of protein sequences using clustalW2 algorithm is very informative in what concerns phylogenetic relations among organisms. Also, it can be helpful to raise the working hypothesis about the effect of observed sequence differences on protein properties. On the other hand, speculations about the effects of the observed differences on actual spatial interactions among amino acid substitutions, even more when they are apart in the linear sequence, are not very reliable and remain only as speculations. The crystal structure of proteins gives reliable information on spatial relationships of amino acids and about pockets, binding sites and also about the electrostatic potential of protein surface. On the other hand protein crystallization and x-ray structure determination remain expensive and technically difficult. Because of that, protein modeling and docking studies became more and more accepted as a method to study protein-ligand interactions. To further test if the hypothesis that the ability of AMP binding to its regulatory site in PFK from different sources of proteins is determined by differences in local charges, experiments of 3D molecular modeling and docking were performed as described in the methods, although we are aware of the limitations of their use in docking studies.

**Fig. 5:**
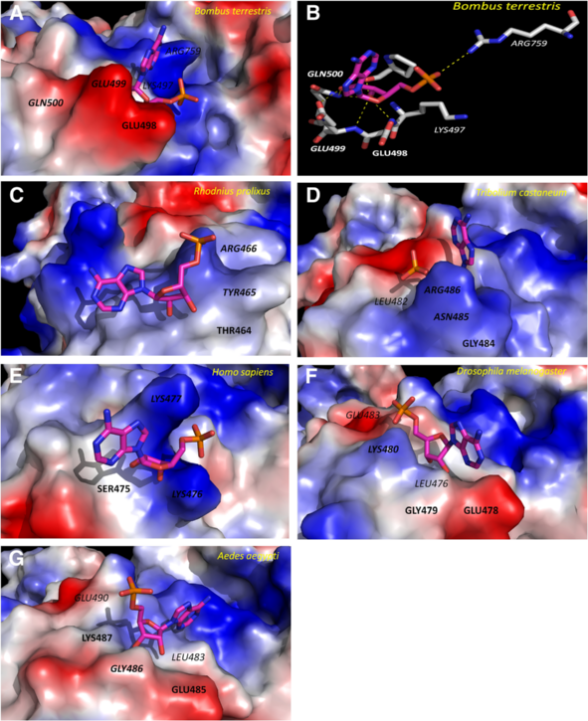
Docking of AMP and Electrostatic surface models. Relevant amino acids to catalysis or AMP stabilization at this pocket are highlighted. Yellow dotted lines in panel B represent the hydrogen bonds and ionic interactions

PFK of the insects *Rhodnius prolixus* and *Bombus atratus* PFK must be included in this study because it was previously shown both are stimulated by AMP [37]. However, we use *Bombus terrestris* PFK sequence, a closely related species, instead of *Bombus atratus* because it does not have its PFK sequence deposited in any databank. It is interesting to see in the alignment of several PFKs that *B. terrestris* PFKs have peculiarities in the region of AMP binding (Fig. 4). The consequences of this sequence change in the electrostatic potential of the local protein surface caused by aspartic acid residues will be better examined in a 3D model. It can be seen in Fig. 5A that AMP succeeds in binding *B. terrestris* PFK. It binds close to the amino acid residues that form the triad Glu498, Glu499 and Gln500, and positively charged amino acid residues Lys497 and Arg759, in the vicinity. The hydroxyl groups from the adenine ring are involved in a hydrogen bonding network with the main-chain amino group of Pro495 and Glu498, with one of the oxygen atoms of the side chain of Glu499 and with the amide group of the side chain of Gln500 (Fig. 5B). Additionally, the anionic phosphate group performs an ionic interaction with Arg759 (Fig. 5B). Altogether, it seems that AMP binding is favoured, since the electrostatic map suggests that, overall, there is enough positive charge at the surface of the protein to counterbalance point negative charges and allow AMP binding and exert stimulatory effects in bombus PFK. To further analyse this explanation, models and docking experiments were performed with other insect PFKs besides *B. terrestris* and human PFK (Fig. 5C-G). The hypothesis of the importance of the electrostatic potential of protein surface at this site to the AMP binding is reinforced as the same could be used to explain the other PFKs analysed.

The apparent insensitivity of AaPFK and *D. melanogaster* PFK to AMP de-inhibition, suggested by Fig. 5C-D, has not been reported previously. Then, we analysed whether this modified PFK regulatory mechanism was actually shared by other dipteran PFKs. As *Culex quinquefasciatus* (vector of Filariasis) and *Anopheles aquasalis* (vector of Malaria) are important disease vectors, any control strategy developed for *A. aegypti* might be applicable to these species also. The PFK activity experiment was expanded to assay the effect of AMP on the PFK activity of *C. quinquefasciatus*, *A. aquasalis* and *Drosophila melanogaster* (an organism considered a model) besides *A. aegypti*. The results presented in Table 3 show that, in all dipterans tested, AMP does not activate PFK, even at inhibitory ATP concentrations. This result reinforces the hypothesis that the negative charge introduced in the putative AMP binding site by the replacement of a glycine by a glutamate could in fact cause those PFKs to be insensitive to AMP. To explain this result, the hypothesis that the lack of an effect of AMP on AaPFK could be related to changes in the AMP binding site was investigated in more detail.

**Table 3:** Effects of AMP on PFK activity in *Diptera*

A MP (μM)	Activity [U (mg protein) ^-1^]			
	*Aedes aegypti*	*Culex quinquefasciatus*	*Anopheles aquasalis*	*Drosophila melanogaster*
0	4.00 ± 0.09	4.36 ± 0.93	5.33 ± 1.19	0.19 ± 0.02
1-1000	3.92 ± 0.21	4.36 ± 0.16	4.79 ± 0.75	0.11 ± 0.05

PFK activity was measured at pH = 7.4, 1 mM F6P, 1 mM ATP with and without (first row) added AMP. The assays were conducted at eight different AMP concentrations in the range of 1-1000 μM. Values are means ± SEM of three independent experiments

In order to suggest an explanation for the putative inability of AMP to bind to AaPFK, but successfully bind to human and bumblebee PFK, comparative molecular modeling and docking studies using the predicted models of Human and *A. aegypti* PFK were performed.

**Fig. 6:**
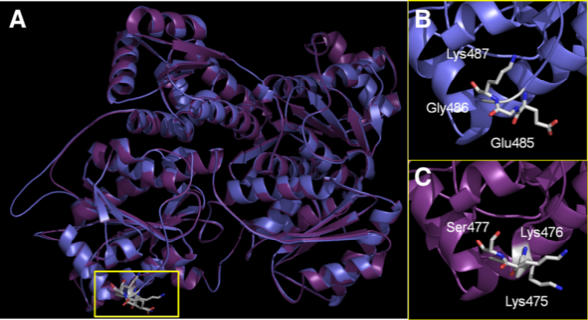
Superimposition of *Aedes aegypti* (*blue*) and *Homo sapiens* (*purple*) PFK models focusing on the AMP binding site. The square marks AMP binding site. Relevant amino acids are highlighted (**b**) and (**c**)

Superimposition of the models of human and AaPFKs (Fig. 6) showed an RMSD of 0.4 Å, which is a very good fit their degree of similarity is considered.

**Fig. 7:**
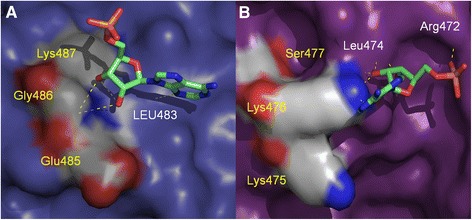
Docking of AMP with *Aedes aegypti* (**a**) and *Homo sapiens* (**b**) PFK models. Relevant amino acids at this pocket are highlighted. Amino acids of the catalytic triad are indicated with yellow legends

A visual inspection of the docking models shows that when the corresponding amino acid residues Glu485 and Lys475 were used as reference sites for docking with *A. aegypti* and human PFK, respectively, the putative observed binding modes are quite different, despite the fitness score values being close (6.99 and 10.09 for *A. aegypti* and human PFKs, respectively) (Fig. 7). The top scored model obtained for AMP and AaPFK places the ribose ring close to Gly486 and Leu483. The hydroxyl groups form a network of hydrogen bonds between the main-chain amino group of Gly486 and the carbonyl group of Leu483. The adenine ring hydrogen bonds to the main-chain amino group of Leu483 (Fig. 6A). The phosphate group, however, was oriented towards the outer part of the binding site, pointing towards the protein solvent-accessible surface. This is a reasonable result because the increased charge density in the putative allosteric AMP binding pocket, caused by the Lys-Glu change from human to AaPFK, is most likely responsible for a better fit of the basic adenosine ring. Additionally, when the best docking model obtained for AMP and human PFK was inspected, the binding mode was inverted in relation to the model obtained with AaPFK. In this case, the adenine ring and the sugar moiety hydrogen bond to Lys476 and Leu474 (Fig. 7B). Again, the presence of basic residues in the triad offers a better electrostatic environment for the anionic phosphate group. It fits in a cavity near and, opposite to, the Lys-Lys-Ser triad, forming an ionic interaction with Arg472 most likely due to a “closed” conformation adopted by Lys476 and Lys477 (Fig. 7B). Finally, there appears to be more room for AMP binding in the human enzyme. The difference in the accommodation of AMP in both PFKs structures led to the calculation of the volume of putative ligand pockets in both enzymes. The comparable pockets comprise Glu485 and Lys475, amongst other amino acid residues (Additional file 3: Figure S3). *A. aegypti* and human PFKs, have volume values of 64.2 and 117.7 Å^3^, respectively. This result corroborates what has been suggested by our docking studies and supports the different binding models observed in both PFKs.

## Conclusions

Dipterans are the most important vectors of the most rapidly spreading diseases around the world. This fact relies especially on the efficient ability of these animals to disperse by flying, which is an action with a high demand for carbohydrate fuel. Here we provide the first study about the unique regulatory mechanism of PFK in flying insects, one of the rate-limiting enzymes of glycolytic flux. By sequence alignment and modeling of dipteran PFKs we propose relevant explanations to their insensitivity to well known effectors, AMP and citrate. A property difference in a key enzyme of a central pathway that has the potential to be targeted by a novel insecticide with the aim to control diseases transmitted by these arthropod vectors was unveiled.

## Additional files

Additional file 1: Figure S1. Alignment of the amino acid sequences of several insects and mammalian PFKs. The putative residues assigned to the binding of effectors/substrates are indicated. α - F6P; β - ATP substrate; χ - ATP inhibitor; δ - citrate; ε - F2, 6BP ϕ - AMP. The sequences shown are from: *Oryctolagus cuniculus* (ORYCU), *Mus musculus* (MUSMU), *Homo sapiens* (HOMSA), *Aedes aegypti*, (AEDAE), *Culex quinquefasciatus* (CULPI), *Anopheles gambiae*, ANOGA), *Drosophila melanogaser* (DROME), *Drosophila virilis* (DROVI), *Drosophila pseudoobscura* (DROPE), *Acyrthosiphon pisum,* (ACYPI), *Tribolium castaneum*, (TRICA), *Pediculus humanus corporis* (PEDHU), *Bombus terretris* (BOMTE), *Rhodnius prolixus* (RPRC). (TIF 9895 kb) Additional file 2: Figure S2. Effect of F2, 6BP on *Aedes aegypti* PFK activity. PFK activity was measured at pH = 7.4, 1 mM F6P, 5 mM ATP at several F2, 6BP concentrations (0.01–50 μM). Values are the means ± SEM of three independent experiments. (TIF 466 kb) Additional file 3: Figure S3. The electrostatic surface of *Aedes aegypti* (A) and *Homo sapiens* (B) PFK models. Red and blue are negatively and positively charged areas respectively. Arrows indicate the entry of the AMP binding pocket. (TIF 955 kb)

## Abbreviations

AaPFK: *Aedes aegypti* phosphofructokinase 1
Ala: Alanine
AMP: Adenosine monophosphate
Arg: Arginine
Asp: Aspartic acid
F1, 6BP: Fructose 1,6 biphosphate
F2, 6BP: Fructose 2,6 biphosphate
F6P: Fructose 6 phosphate
Glu: Glutamic acid
Gly: Glycine
Leu: Leucine
Lys: Lysine
PFK: Phosphofructokinase 1
Ser: Serine
Thr: Threonine
