# Supplementary figures and images for: Unique PFK regulatory property from some mosquito vectors of disease, and from Drosophila melanogaster
Source: Parasit Vectors. 2016 Feb 25;9:107. doi: 10.1186/s13071-016-1391-y (PMC4766633; doi:10.1186/s13071-016-1391-y)

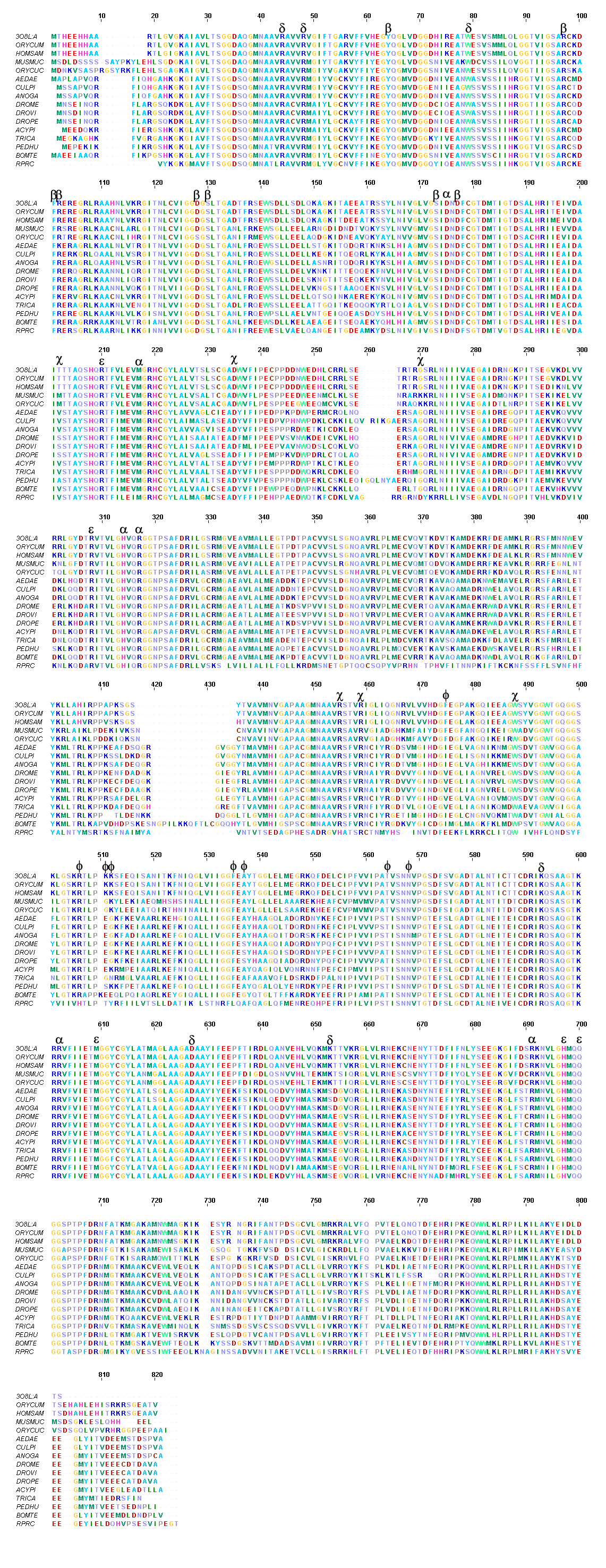

Supplement: Additional file 1: Figure S1. — Alignment of the amino acid sequences of several insects and mammalian PFKs. The putative residues assigned to the binding of effectors/substrates are indicated. α - F6P; β - ATP substrate; χ - ATP inhibitor; δ - citrate; ε - F2, 6BP ϕ - AMP. The sequences shown are from: Oryctolagus cuniculus (ORYCU), Mus musculus (MUSMU), Homo sapiens (HOMSA), Aedes aegypti, (AEDAE), Culex quinquefasciatus (CULPI), Anopheles gambiae, ANOGA), Drosophila melanogaser (DROME), Drosophila virilis (DROVI), Drosophila pseudoobscura (DROPE), Acyrthosiphon pisum, (ACYPI), Tribolium castaneum, (TRICA), Pediculus humanus corporis (PEDHU), Bombus terretris (BOMTE), Rhodnius prolixus (RPRC). (TIF 9895 kb) [file 13071_2016_1391_MOESM1_ESM.tif]

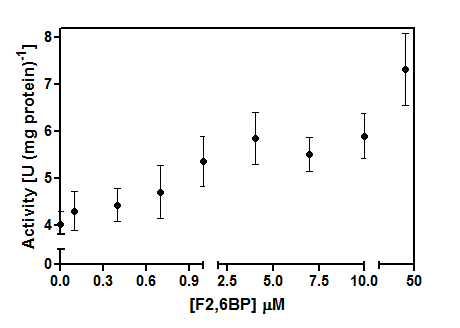

Supplement: Additional file 2: Figure S2. — Effect of F2, 6BP on Aedes aegypti PFK activity. PFK activity was measured at pH = 7.4, 1 mM F6P, 5 mM ATP at several F2, 6BP concentrations (0.01–50 μM). Values are the means ± SEM of three independent experiments. (TIF 466 kb) [file 13071_2016_1391_MOESM2_ESM.tif]

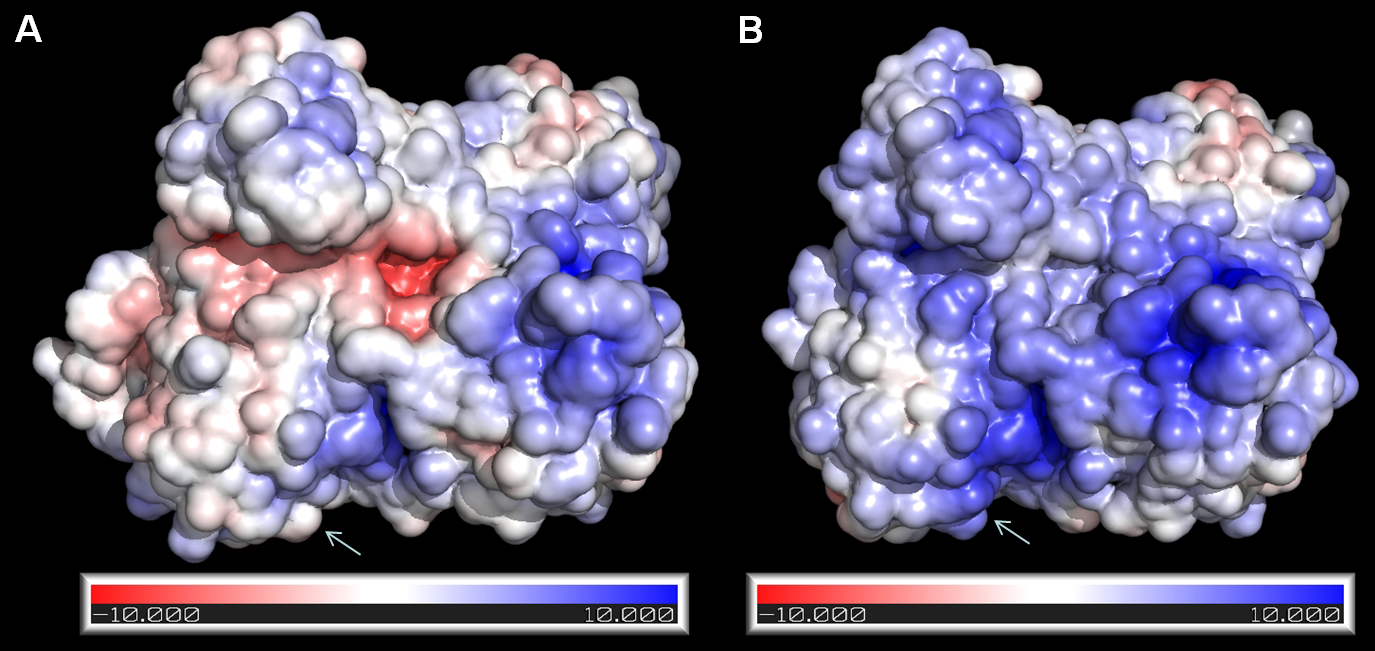

Supplement: Additional file 3: Figure S3. — The electrostatic surface of Aedes aegypti (A) and Homo sapiens (B) PFK models. Red and blue are negatively and positively charged areas respectively. Arrows indicate the entry of the AMP binding pocket. (TIF 955 kb) [file 13071_2016_1391_MOESM3_ESM.tif]
